# Drug Interactions With New Synthetic Opioids

**DOI:** 10.3389/fphar.2018.01145

**Published:** 2018-10-11

**Authors:** Clara Pérez-Mañá, Esther Papaseit, Francina Fonseca, Adriana Farré, Marta Torrens, Magi Farré

**Affiliations:** ^1^Clinical Pharmacology Unit, Hospital Universitari Germans Trias i Pujol (HUGTP-IGTP), Badalona, Spain; ^2^Department of Pharmacology, Therapeutics and Toxicology, Autonomous University of Barcelona, Barcelona, Spain; ^3^Drug Addiction Unit, Institute of Neuropsychiatry and Addictions, Hospital del Mar Medical Research Institute, Barcelona, Spain; ^4^Department of Psychiatry and Legal Medicine, Autonomous University of Barcelona, Barcelona, Spain

**Keywords:** interaction, fentanyl, fentanyl analogs, new synthetic opioids, new psychoactive substances

## Abstract

Fentanyl, fentanyl analogs, and other new synthetic opioids (NSO) have burst onto the illegal drug market as new psychoactive substances (NPS). They are often sold as heroin to unsuspecting users and produce euphoria through their agonist action on μ- opioid receptors. Their high consumption, often combined with other substances, has led to multiple intoxications during recent years. In some countries, such as the United States, the consumption of opioids, whether for medical or recreational purposes, has become epidemic and is considered a public health problem. Fentanyl analogs are more potent than fentanyl which in turn is 50 times more potent than morphine. Furthermore, some fentanyl analogs have longer duration of action and therefore interactions with other substances and medicines can be more serious. This review is focused on the potentially most frequent interactions of opioid NPS taking into account the drugs present in the reported cases of poly-intoxication, including other illegal drugs of abuse and medication. Substances involved are mainly antidepressants, antihistamines, antipsychotics, benzodiazepines, analgesics, anesthetics, psychostimulants, other opioids, alcohol, and illegal drugs of abuse. The interactions can be produced due to pharmacokinetic and pharmacodynamic mechanisms. Naloxone can be used as an antidote, although required doses might be higher than for traditional opioid intoxications. It is crucial that doctors who habitually prescribe opioids, which are often misused by patients and NPS users, be aware of designer opioids’ potentially life-threatening drug-drug interactions in order to prevent new cases of intoxication.

## Introduction

### New Synthetic Opioids as New Psychoactive Substances (Opioid NPS)

New psychoactive substances (NPS) are compounds designed to mimic classical illegal recreational drugs (Tracy et al., 2017). Synthetized by slightly tweaking the molecular structure of an existing illegal drug or a legally prescribed medication, they have spread rapidly in recent years. As new chemical substances they are considered legal by default until outlawed which, in most cases, may take several years after reaching the market, for this reason, they are sometimes referred to as “legal highs.” Some NPS have also been called “research chemicals,” psychoactive substances which have played little or no role in scientific and medical studies.

According to the United Nations Office on Drugs and Crime (UNODC) NPS are substances of abuse, either in a pure form or as a preparation, that do not come under the 1961 Single Convention on Narcotic Drugs or the 1971 Convention on Psychotropic Substances, but which may pose a public health threat (United Nations Office on Drugs and Crime [UNODC], 2018a). Not all NPS are new substances, some were synthetized 40 years ago, but all of them have recently reached the market. NPS are usually obtained through internet or specialist establishments (head shops), sold under a broad range of names and brands, and generally consumed by experienced drug users.

The chemical diversity of these products and their exponential increase complicates monitoring. Among all the various reported NPS, the opioid group does not, however, include a large amount of chemical substances in comparison with others. Between 2009 and 2017, a total of 779 NPS were reported to the UNODC with the majority from 2016, 34 were opioids, including 26 fentanyl analogs (United United Nations Office on Drugs and Crime [UNODC], 2017; United Nations Office on Drugs and Crime [UNODC], 2018c). UNODC registered 72 first-time NPS in 2017, and according to the most recent European data, a total of 51 NPS were detected in 2017, 13 of which were new opioids (European Monitoring Centre for Drugs and Drug Addiction [EMCDDA], 2018b).

Moreover, 16.0% of the total sample (13000 respondents) of the Global Drug Survey (2018) reported lifetime use of NPS, and 5% reported previous 12-month use. In addition, the drug effects that the NPS attempted to mimic were opioid-like in 8.9% of cases (Global Drug Survey, 2017).

In Europe, 3% of students aged 15–16 years admitted to previous year use of NPS and 4% lifetime use in 2015 (European School Survey Project on Alcohol and Other Drugs, 2015).

The term opioid applies to any substance, whether endogenous or synthetic, that produces morphine-like effects. Opiates are restricted to the natural plant alkaloids, such as morphine, codeine, thebaine, and many semisynthetic derivatives. Additionally, the term novel or new synthetic opioids (NSO) has been used to refer to emerging fentanyl analogs and non-fentanyl compounds with other chemical structures, all of them included in the group of NPS opioids.

In an online survey conducted in 2015 with 619 NPS international users, among the 1551 NPS consumed 3.3% were opioids. The most common drugs in this group were Kratom (56.6%), AH-7921 (9.4%), and 0-desmethyltramadol (5.7%) (Soussan and Kjellgren, 2016).

There is a growing supply and consumption of illicitly manufactured synthetic opioids such as fentanyl, fentanyl analogs, and NSO belonging to other structural groups. The following are non-fentanyl analogs: U-47700, U-448800, U-77891, U-50488, U-51754 AH-7921, MT-45, and O-desmethyltramadol (Prekupec et al., 2017; Ventura et al., 2018).

Regarding legalization, fentanyl is a Schedule II drug under the Controlled Substances Act in the United States (Drug Enforcement Administration, 2018). Substances and chemicals included in schedule II are defined as drugs with a high potential for abuse, with consumption potentially leading to severe psychological or physical dependence. Schedule II also encompasses pharmaceutical fentanyl analogs. Some non-pharmaceutical fentanyl analogs are in Schedule I, which contains non-medical substances with high abuse potential. On the other hand, fentanyl and its pharmaceutical analogs are Schedule I substances according to the United Nations Single Convention on Narcotic Drugs 1961, amended in 1972 (substances that are highly addictive and liable to abuse). In turn, some non-pharmaceutical analogs are schedule I–IV substances, while new compounds are being progressively incorporated into the lists (United Nations Office on Drugs and Crime [UNODC], 2018b).

### Epidemiology of Opioid Overdose Crisis

A worrisome increase in overdose deaths involving synthetic opioids (licit and illicit fentanyl and its analogs, and also other NSO) has been detected mainly in the United States (US) in recent years and is spreading to other countries such as Canada, Australia, and Japan, in addition to Europe.

In the 1970–1980s, synthetic opioids, such as fentanyl and its analogs, first appeared combined with heroin (China White, Tango and Cash, synthetic heroin). Since 2014 resurgence of this phenomenon has been observed, in this case with fentanyl and fentanyl analogs produced clandestinely, and also with the introduction of NSO not intended for medical use.

Non-medical use (or misuse) of a drug refers to the use, whether obtained by prescription or otherwise, other than in the manner or for the time period prescribed, or by a person for whom the drug was not prescribed (United United Nations Office on Drugs and Crime [UNODC], 2017). With respect to opioids, parallel to their dramatic increase in prescription has been their non-medical use over the last two decades in the US and Canada. It is noteworthy, however, that although opioid prescriptions have decreased recently in these countries (van Amsterdam and van den Brink, 2015; Piper et al., 2018) opioid overdose death rates continue increasing. These epidemiological findings can be explained by the rise in illicitly manufacturated fentanyl (IMF) and NSO contaminating heroin and counterfeit pain pills, leading to an epidemic of poisonings.

In Europe, the medical use of opioids also increased during the previous decade although at a slower rate, while non-medical use has been rarely reported (van Amsterdam and van den Brink, 2015). Furthermore, changes in prescribed recommended doses, or routes of administration, raise overdose risk. As an example, some individuals misuse fentanyl by extracting it from patch formulations and then injecting it, without knowing the exact dose taken (Tharp et al., 2004).

The increase in opioid overdoses can thus be explained by a rise in the consumption of synthetic opioids from different origins and by various collectives. They can be illicitly manufactured or diverted from pharmaceutical fentanyl or derivatives, and deceased subjects may be heroin addicts looking for a substitute, patients previously treated for pain, and NPS users. The contribution of each collective depends on the country and year, and incomplete data hinders the establishment of exact figures. The situation is evolving, nowadays overdoses driven by or involving illicitly manufactured fentanyl and fentanyl derivatives are the most common, particularly in the US and Canada.

Furthermore, constant changes in NPS opioids available on the market, and analytical difficulties in identifying them (common toxicology screens do not detect NPS opioids that have little structural similarities to morphine and other commonly tested opioids), make it difficult to blame fentanyl analogs and other NSO for overdoses, as a result, underreporting is probable (Pichini et al., 2017; Baumann and Pasternak, 2018).

#### United States

The opioid epidemic or opioid crisis initially focused on extended-release prescription opioids. Later, heroin (greater supply and use) and IMF (mixed with heroin or alone) expanded into the context of widespread opioid prescription misuse (O’Donnell et al., 2017a,b; Schnoll, 2018).

Opioids accounted for 66.4% of all overdose deaths in the US in 2016 (Seth et al., 2018). The recent rise in synthetic opioid overdoses is largely due to IMF, and fentanyl analogs are implicated in 17% of fentanyl-related deaths (Prekupec et al., 2017). As an example, between January and February 2017, in Ohio among 281 deaths, 90% tested positive for fentanyl, 48% for acrylfentanyl, 31% for furafentanyl, 8% for carfentanil, 6% for heroin, and 23% for pharmaceutical opioids (Daniulaityte et al., 2017).

Data from October 2017 report 68,400 overdose deaths in the previous year in the US: 40,149 related to opioids, 17,027 due to synthetic opioids analgesics other than methadone (fentanyl, tramadol), 14,984 due to heroin, 14,072 due to natural opioid analgesics (morphine and codeine) and semisynthetic opioids (oxycodone, hydrocodone, hydromorphone, and oxymorphone), and 3,343 due to methadone (Ahmad et al., 2018).

Regarding trends in medical use, since 2011, opioid prescription has been decreasing, with the exception of buprenorphine, employed to treat opioid use disorder. These data reflect favorable results from reduction prescription polices. The percentage change in fentanyl prescription in 2016 was -8.9% relative to 2015. The most prescribed drugs were oxycodone, followed by hydrocodone, morphine, and codeine (Piper et al., 2018).

Another important adopted measure has been to reduce the number of days in treatment, as it correlates with the maintenance of opioid treatment 1 year later. In fact, 6% of those with at least 1 day of treatment were on opioids 1 year later, while it increased to 30% in those with at least 31 prescription days (Shah et al., 2017).

#### Canada

Until 2012 opioid deaths were mainly related with medically prescribed opioids (modified release oxycocone formulations). Opioid-related overdose deaths rose from 2010 to 2016, and in 2016 fentanyl products represented from 60 to 2% of all opioids depending on the province (Fischer et al., 2018). In the same year there were 2,861 apparent opioid-related deaths in Canada. From January to June 2017, 74% involved fentanyl or its analogs, compared to 53% in 2016, and 74% of deaths occurred among males and 28% among individuals aged 30–39 years. No distinction was made between pharmaceutical and non-pharmaceutical opioids (Public Health Agency of Canada, 2017).

#### Australia

The most common class of drug identified in toxicology reports about drug-induced deaths is opioids. From 2011 to 2015, 3,601 individuals died from overdoses due to an opioid (1.6-fold increase from 2001 to 2005). In this period accidental deaths from oxycodone, morphine, and codeine were responsible for most opioid-related deaths, followed by heroin, fentanyl, tramadol, and pethidine. A significant increase was, however, observed in fatal overdoses due to fentanyl, with an 8-fold increase over 10 years. In 2015 70% of those who died from overdose were males aged 30–59 years (Penington, 2017).

There was a 4-fold increase in pharmaceutical opioid use between 1990 and 2014. Non-medical use of pharmaceutical opioids increased over time among individuals who injected drugs. Extra-medical use of fentanyl is suspected as only fentanyl deaths increased significantly taking into account prescriptions (Roxburgh et al., 2017).

#### Europe (EU)

Heroin remains the most commonly used illicit opioid, but a number of sources suggest that licit synthetic opioids are increasingly misused. Opioids reported by treatment centers include misused methadone, buprenorphine, fentanyl, codeine, morphine, tramadol, and oxycodone. In 2016, in 18 EU countries more than 10% of all opioid clients entering specialized services presented opioids other than heroin (European Monitoring Centre for Drugs and Drug Addiction [EMCDDA], 2018a).

Recently, an overall increase in opioid-related overdose deaths, as well as rising reports of problems with NSO, has been detected. The average prevalence of high-risk opioid use among adults (15–64 years) is estimated at 0.4 % of the EU population, the equivalent of 1.3 million high-risk opioid users (European Monitoring Centre for Drugs and Drug Addiction [EMCDDA], 2017). Opioids are found in 81% of fatal overdoses. The role that synthetic opioids play in overdose deaths is difficult to quantify, but in many countries these substances are gaining importance, and in a few predominate. Overall, 25 new opioids have been detected since 2009, including 18 fentanyls. (European Monitoring Centre for Drugs and Drug Addiction [EMCDDA], 2017).

In Estonia an endemic problem with fentanyl has existed since the early 2000s with high rates of overdose in comparison with other EU countries. In 2010–2012 both fentanyl and 3-methylfentanyl were marketed as a replacement for heroin in EU countries affected by heroin shortages (e.g., Bulgaria, Slovakia). More recently (2006–2012), Germany, Finland, and the United Kingdom reported new outbreaks of fentanyl-related deaths (Mounteney et al., 2015).

Between 2015 and 2017 intoxications due to fentanyl and its analogs occurred mostly in Sweden, but also in Hungary, Belgium, Switzerland, Poland, and the United Kingdom (Pichini et al., 2018).

Although in the EU, in contrast to the US, very few patients require specialized drug treatment for addiction to opioid pain medication, underreporting cannot be dismissed. In fact, among young people 4% of students aged 15–16 years reported lifetime use of painkillers to get high (European School Survey Project on Alcohol and Other Drugs, 2015).

Data from the Early Warning System reported 250 deaths in 2016–2017 linked to fentanyls. It was the first time new opioids were the single largest group of new substances to appear. Nowadays, 38 new opioids are being monitored, 28 of them highly potent fentanyl analogs (European Monitoring Centre for Drugs and Drug Addiction [EMCDDA], 2018a).

#### Japan

Due to the low consumption rate of opioids in Japan they are not among the leading causes of overdose-related deaths which are benzodiazepines and barbiturates (Okumura et al., 2017). However, several cases of intoxication with fentanyl analogs have been recently reported (Takase et al., 2016; Yonemitsu et al., 2016).

### Epidemiology of Opioid Consumption With Medication and Drugs of Abuse

In most opioid overdoses other substances are found or involved. As commented before, NPS opioids cannot be easily detected in routine screen tests, however, these tests can be helpful in detecting concomitant use of other drugs.

In 2016, at least half the people who died from an opioid overdose in the US were taking fentanyl, and 57% of those with positive tests for fentanyl or analogs were also positive for other drugs such as heroin (O’Donnell et al., 2017b). In Canada approximately 82% of apparent opioid-related deaths from 2016 to 2017 also involved one or more type of non-opioid substances (mainly alcohol, benzodiazepines, cocaine, and W-18) (Public Health Agency of Canada, 2017).

A study conducted in Boston compared toxicological findings in drug overdose fatalities due to illicit fentanyl with data from accidental fatalities (licit use). There were 55 cases of illicit use, 26 of licit use, and 26 of indeterminate use. Deaths associated with illicit use occur in younger people (40 *vs.* 62 years) with higher fentanyl concentrations (17.1 vs.4.4 ng/ml) and more frequent cocaine co-intoxication (65 vs. 12%). The presence of other opioids was higher in the group of licit fentanyl (81 vs. 55%). In illicit users the most common opioids were morphine, oxycodone, and methadone, while for licit ones it was mainly morphine. Ethanol, cannabinoids, diazepam, citalopram, and diphenhydramine were each detected at rates greater than 10% in the illicit fentanyl cases (Hull et al., 2007).

Other substances involved in NSO overdoses, taking into account the published cases of intoxication (Hull et al., 2007; Lee et al., 2016; McIntyre et al., 2017; Zawilska, 2017), are listed in **Table 1**.

**Table 1:** Other substances involved in new synthetic opioid overdoses, taking into account the published cases of intoxication (Hull et al., 2007; Lee et al., 2016; McIntyre et al., 2017; Zawilska, 2017).

Group	Substances
Anticholinergics	Dicycloverine
Antihistamines	Hidroxycine, prometazine, diphenhydramine, chlorphenyramine, doxylamine
Antiinflammatory or analgesic drugs	Ibuprofen, acetaminophen, salicylic acid, naproxen
Antidepressants	Fluoxetine, mirtazapine, citalopram, sertraline, amitriptyline, venlafaxine
Antipsychotics	Clorprormazine, risperidone, quetiapine, olanzapine, doxepin, promethazine
Anticonvulsants	Pregabalin, clonazepam, gabapentin, carbamazepine
Barbiturates	Phenobarbital
Benzodiazepines	Diazepam, alprazolam, nordiazepam, chlordiazepoxide, etizolam, lorazepam
Stimulants	Ephedrine, cocaine (benzoylecgonine), amphetamine, nicotine (cotinine), mephedrone
Other opioids	Morphine, oxycodone, 6-acetylmorphine, methadone hydrocodone, meperidine (pethidine), tramadol,6-acetylmorphine, propoxyphene, hydromorphone, codeine, tramadol, buprenorphine, heroin, dextrometorphan,
Other drugs of abuse	Ethanol, delta-9-tetrahidrocannabinol, synthetic cannabinoids, ketamine,
Other substances	Levamisole, lidocaine

Illicitally manufacturated fentanyl and its analogs can be stand-alone products, low cost additives to increase the potency of other illicit drugs including heroin, cocaine, and methamphetamine, or sold as counterfeit medicines, such as oxycodone, hydrocodone, and alprazolam.

On the other hand, patients treated with opioids for pain frequently have other comorbidities and 67% of them receive other prescription drugs. A retrospective analysis showed that among patients with chronic back pain on long-term opioids, prevalence of drug–drug interactions (DDI) was 27%. Metabolic DDI involving cytochrome P450 (CYP450) were among the most common (Pergolizzi and Raffa, 2017). Furthermore, in a systematic review of opioid-related problems including 105 publications, 30% describes opioid-associated DDI (Butts and Jatoi, 2011).

## Classification

For the purpose of this review the included NPS opioids will be fentanyl, fentanyl analogs, and NSO that are non-fentanyl analogs.

### Fentanyl

Fentanyl or *N*-(1-(2-phenethyl)-4-piperidinyl-*N*-phenyl-propanamide is a piperidine derivative used in severe pain and anesthesia. It was originally synthesized by Janssen Pharmaceutical in 1974. There are several formulations of pharmaceutical fentanyl: solutions for injection, transdermal patches and lozenges, and buccal tablets. It produces analgesia, drowsiness, and euphoria, the last effect less than heroin and morphine. Common side effects are nausea, dizziness, vomiting, fatigue, headache, constipation, and edema. Overdose causes respiratory depression, miosis, and sedation, and can lead to cardiac arrest.

Prescribed fentanyl can be “diverted” in a number of ways: it can be obtained inappropriately through the individual’s own profession (e.g., healthcare workers), used for a non-medically intended purpose by someone who has been prescribed it, or by employing another person’s prescription.

Non-prescribed fentanyl is misused by injection, oral ingestion of lozenges, and patches. Fentanyl powder and patches are also smoked and snorted. Furthermore, patches can be taken orally (sublingually) by sectioning them into doses which requires a solvent such as overproof ethanol for sufficient absorption (European Monitoring Centre for Drugs and Drug Addiction [EMCDDA], 2018c). IMF is also sometimes mixed with other drugs to increase potency.

### Fentanyl Analog or Derivatives

Fentanyl analogs have been developed by adding various substituents to the basic molecule to modify potency. Those that have recently appeared are generated by modification or replacement of the fenanyl propionyl chain or replacement of the ethylphenyl moiety. Furthermore, existing variants have been substituted with chloro, fluoro or methoxy substituents at the *N*-phenyl ring. Some fentanyl analogs are medically used but most lack medical indication.

#### Pharmaceutical Fentanyl Derivatives

The fentanyl analogs with medical use (anesthesia) are sufentanil, alfentanil, and remifentanil. Carfentanyl is only approved for veterinary issues.

#### Non-pharmaceutical Fentanyl Derivatives or “Designer” Fentanyls

It should be noticed that the term non-pharmaceutical fentanyl is not only restricted to fentanyl analogs without medical use. It also includes illicitly produced pharmaceutical fentanyls (described in the previous section).

There is a long list of non-pharmaceutical fentanyl analogs. Those reported in published reviews of cases of intoxication (Suzuki and El-Haddad, 2017; Zawilska, 2017; Pichini et al., 2018) are the following: alpha-methyl-fentanyl, 3-methylfentanyl, acetylfentanyl, butyrylfentanyl, beta-hydroxy-thio-fentanyl, 4-fluorobuyrylfentanyl, furanylfentanyl, ocfentanil, acrylyolfentanyl, 4-methoxibutyrylfentanyl, tetrahydr ofuranfentanyl, beta-hydroxytihiofentanyl, para-fluoroisobut yrylfentanyl, cyclopentyfentanyl, 4-fluoroisobutyrylfentanyl, and 4-chloroisobutyrylfentanyl.

Fentanyl analogs reported to the UNODC Early Warning Advisory, 2012–2016 (United United Nations Office on Drugs and Crime [UNODC], 2017) are the following: 3-fluorofentanyl, 4-fluorobutyrfentanyl, 4-methoxybutyrfentanyl, acetylfentanyl, acrylfentanyl, beta-hydroxy-thiofentanyl, butyrfentanyl, despropionylfentanyl, despropionyl-2-fluorofentanyl, furanylfentanyl, isobutyrfentanyl, (iso)butyr-F-fentanyl *N*-benzyl analog, methoxyacetylfentanyl, ocfentanil, para-fluoroisobutyrfentanyl, tetrahydrofuranylfentanyl, and valerylfentanyl.

### New Synthetic Opioid Non-fentanyl Analogs

The following NSO are structurally unrelated to fentanyl and have been reported in cases of intoxication: U-47700, AH-7921, MT-45, bromadoline, U-50488, U-51754, U-77891 (Suzuki and El-Haddad, 2017; Solimini et al., 2018). Regarding their chemical structure, U-47700 (from AH-7921/doxylam), U-448800, and U-77891 are benzamides, U-50488 and U-51754 are acetamides, and MT-45 is a piperazine (Solimini et al., 2018).

Other non-fentanyl analogs included in the group of NPS opioids are desomorphine and *O*-desmethyltramadol, (Ventura et al., 2018). Desomorphine is a morphine analog and a component of krokodil, a homemade opioid synthetized from codeine.

## Clinical Pharmacology

### Mechanism of Action and Pharmacodynamics

Most NSO have mechanisms of action and effects similar to established opioids. Their main effects are mediated through activation of μ-opioid receptors (analgesia, respiratory depression, euphoria, miosis, decreased intestinal motility, sedation, addiction, and dependence). As an example, U-47700 is a potent μ-opioid receptor agonist, AH-7921 is an agonist of μ and κ receptors, and MT-45 is an agonist of μ, κ and δ opioid receptors. Interestingly antinociceptive effects of U-47700, MT-45 and butyrylfentanil have been studied in mice. These compounds act as agonists of murine μ-opioid receptor 1 but *in vitro* finding affinity does not predict *in vivo* potency (Baumann et al., 2018).

Some others, however, have different mechanisms of action. U-50488 is mainly a κ-opioid receptor agonist that has been studied in animal models as an analgesic, antitussive, diuretic, and anticonvulsant. Its side effects include dysphoria and hallucinations, and it has been reported to present μ-opioid receptor antagonist effects. U-51754 is not as selective for KOR as the previous one (Solimini et al., 2018; Ventura et al., 2018).

New synthetic opioids overdoses are characterized by the presence of the following triad: respiratory depression, stupor-sedation, and miosis. The major cause of death is respiratory failure. Additional clinical features include bradycardia, cyanosis, hypotension, pulmonary edema, ileus, nausea, vomiting, and pruritus. Atypical characteristics in overdoses with fentanyl and analogs have been described such as immediate blue discoloration of the lips, gurgling sounds with breathing, foaming at the mouth, confusion, seizure-like activity, and chest wall rigidity. For MT-45 bilateral hearing loss, low miotic effect, hair depigmentation and loss, folliculitis, dermatitis, dry eyes, liver enzyme alteration, leukonychia striata on the nails, and cataracts have been observed (Armenian et al., 2017; Prekupec et al., 2017; Solimini et al., 2018; Ventura et al., 2018). Desomorphine when injected can cause thrombophlebitis, ulcerations, and gangrene.

Fentanyl withdrawal symptoms include anxiety, diarrhea, shivers, abdominal cramps, and sweating. Addiction and tolerance are also quickly achieved with repeated use of NSO; U-50488 abuse potential is, however, unknown (Ventura et al., 2018).

*In vivo* potency is usually measured by analgesic activity in rodent species. Regarding potency, fentanyl is 50–100 fold more potent than morphine and 24–40 fold more potent than heroin. The enhanced *in vivo* potency of fentanyl is most likely related to its higher lipophilicity and brain penetration when compared to morphine. Some fentanyl analogs have higher potency than fentanyl. Sufentanil and carfertanil potency in relation to morphine are 500–1,000 and 10,000 fold, respectively. Non-pharmaceutical analogs have higher potencies than morphine: butyryl-fentanyl (1.5-7), acetylfentanyl and 4-fluorobutyrfentanyl (15.7), alpha-metyl-fentanyl (56.9), octafentanil (90), 3-methyl-fentanyl (48.5-569), and beta-hydroxy-3-methylfentanyl (6,300).

Several NSO with other chemical structures have lower potencies than fentanyl: 1.7 for AH-7921, 3.5 for MT-45, and 7.5 times for U-47700 in relation to morphine (Prekupec et al., 2017; Suzuki and El-Haddad, 2017). Desomorphine is a morphine analog 10 times more potent than morphine. In turn, *O*-desmethyltramadol is 2–4 times more potent than tramadol itself (Dickman, 2007), which is ten times less potent than morphine.

### Pharmacokinetics

Fentanyl can be administered through intravenous, oral, epidural, intranasal, intrathecal, and transdermal routes. Different routes of administration have also been reported for fentanyl analogs and other NSO: oral, sublingual, nasal insufflation, nasal spray, inhalation, rectal, and intravenous injections. As an example, acetylfentanyl can be smoked, snorted and intravenously injected; for butyrylfentanyl, rectal, nasal, intravenous, transdermal, and sublingual routes have been reported; and AH-7921 administration can be nasal, intravenous, insuflated, and oral (Ventura et al., 2018). Additionally E-liquids containing fentanyls can be vaped using electronic cigarettes (European Monitoring Centre for Drugs and Drug Addiction [EMCDDA], 2018a).

The half-life and duration of fentanyl effects depend on the route of administration. Fentanyl elimination half-life is 7 h for buccal and transmucosal routes, 219 min for intravenous, and 2.63–11.7 h for transdermal (Food and Drug Administration [FDA], 2018). Fentanyl pharmaceutical derivative half-lives are 90–111 min for alfentanil, 164 min for sufentanil, and 3–10 min for remifentanil (Food and Drug Administration [FDA], 2018). No pharmacokinetic data are available for non-pharmaceutical analogs and other NSO as they have not been experimentally administered to humans.

Most opioids are lipophilic, consequentially fentanyl and its analogs can pass easily through membranes, including the blood brain barrier. Fentanyl analogs have closer chemical structures to fentanyl than morphine so low oral bioavailability is predicted. In the case of intranasal fentanyl bioavailability is 89%, while oral transmucosal administration has a bioavailability of 50% (Armenian et al., 2017).

Fentanyl has a rapid onset (2–5 min) and a short duration of action (around 1–4 h) for transmucosal, insuflated, and buccal routes, and longer for transdermal ones (48–72 h). Fentanyl pharmaceutical derivatives such as alfentanil, sufentanil, and remifentanil are all administered by intravenous route in anesthesia and have very short onset and duration of action. For NSO that are non-fentanyl analogs data from self-administration describe longer effects (6–8 h for AH-7921, 4–6 h for MT-45, and 5–7 h for U-47700 by oral route). However, for U-47700 via the intravenous route the onset is 1 min and the duration of action 1–2 h, producing a strong urge for redosing (Zawilska, 2017; Ventura et al., 2018).

Chemical reactions metabolizing a drug generally make it more water soluble and can be classified in phase I (hydrolysis, oxidation, reduction) and phase II reactions (conjugation with glucuronide, sulfate, glycine, and glutation). Opioids undergo phase I metabolism by the cytochrome P450 system, phase II metabolism by conjugation, or both. Phase I metabolism of opioids mainly involves the CYP3A4 and CYP2D6 enzymes, and glucuronidation is catalyzed by uridine diphospate glucuronosyltransferase (UGT). Most oxidative metabolism of opioids is performed in the liver, but also in enterocytes responsible for first-pass metabolism mediated by CYP3A enzyme family (Gudin, 2012).

In the following paragraphs routes of metabolism for morphine, heroin, fentanyl and its analogs, and other NSO are described.

Morphine has minimal phase I metabolism, and follows glucuronidation via UGT2B7. Its metabolism may be altered by interactions with other drugs that are either substrates or inhibitors of this enzyme. Morphine is glucuronidated to two active metabolites: morphine-6-glucuronide and morphine-3-glucuronide, and also undergo minor routes of metabolism, including *N*-demethylation to normorphine or normorphine 6-glucuronide, diglucuronidation to morphine- 3, 6-diglucuronide, and formation of morphine ethereal sulfate. A minor conversion to hydromorphone has also been described (Smith, 2009).

Heroin (diacetyl-morphine or diamorphine), in turn, is rapidly deacetylated to 6-monoacetylmorphine (6MAM), then further metabolized into morphine. Both 6MAM and morphine are bioactive metabolites. In fact recent evidence suggest that 6MAM could have a major role mediating heroin effects (Gottås et al., 2013). Hydrolysis of heroin and 6MAM is thought to be catalyzed by different types of esterases (Rook et al., 2006).

Fentanyl is mainly metabolized via the CYP3A4 isoenzyme in the liver and intestinal mucosa to norfentanyl through *N*-dealkylation (norfentanyl has not been reported to be pharmacologically active in animal studies). Less than 1% is metabolized to despropionylfentanyl, hydroxyfentanyl, and hydroxynorfentanyl which also lack clinically relevant activity. It has no phase II metabolism. A small amount (5–10%) is renally and fecally cleared (Smith, 2009; Food and Drug Administration [FDA], 2018).

Alfentanil follows piperidine and amide *N*-dealkylation to noralfentanil and *N*-phenylpropionamide through CYP3A4 (Klees et al., 2005; Kharasch et al., 2011). It can be used as an *in vivo* probe for hepatic and first-pass CYP3A activity, although midazolam is the reference substance. In the case of alfentanil, pupil diameter change is a surrogate for plasma concentrations, leading to noninvasive assessment of CYP3A. Sufentanil and carfentanyl are also primarily metabolized via CYP3A4 generating *N*-dealkylated metabolites (Guitton et al., 1997; Feasel et al., 2016). Remifentanil, in turn, is mainly metabolized through non-CYP enzymes. It follows rapid hydrolysis through blood esterases and the *N*-dealkylated metabolite is minor (Bürkle et al., 1996).

Little information is available about the metabolism of non-medical fentanyl analogs. Alpha-methylfentanyl, 3-methylfentanyl, and isofentanyl are also metabolized to the nor-metabolite, in a similar manner to fentanyl (Watanabe et al., 2017). 3-methylfentanyl is metabolized in rats through CYP2D6, CYP2C19, CYP3A4, and CYP3A5 (Meyer et al., 2012).

The metabolic profile of acetylfentanyl, acrylfentanyl, 4-fluoro-isobutyrylfentanyl, and furafentanyl has also been studied *in vitro* with human hepatocytes and human urine samples after consumption. The first three were predominantly metabolized by *N*-dealkylation like fentanyl. In addition, each also has a hydroxyethyl and hydroxymethoxy metabolite. Additionally, human live microsomal and *in vivo* studies in rodents have demonstrated that acetylfentanyl is converted to acetylnorfentanyl through cytochrome P450 (Patton et al., 2014).

On the other hand, furanyfentanyl major metabolites are not generated by *N*-dealkylation. It undergoes amide hydrolysis with/without hydroxylation and dihydrodiol formation, while the nor-metabolite was undetected in urine samples (Watanabe et al., 2017).

Butyrylfentanyl metabolism was studied *in vitro* in liver microsomes and with recombinant cytochrome P450 enzymes and *in vivo* in urine samples. It also has different metabolites (carboxybutyrfentanyl, hydroxybutyrfentanyl, norbutyrfentnayl, and desbutyrfentanyl) generated by hydroxylation and carboxilation of the butyryl side chain in this case, and mainly CYP2D6 and 3A4 are involved (Steuer et al., 2017).

Non-fentanyl NSO analogs such as AH-7921 follow demethylation, as U-4770, for which *N*-desmethyl,*N,N*-didesmethyl, desmethylhydroxy and *N,N*-didesmethylhydroxy metabolites in urine have been detected (Wohlfarth et al., 2016; Jones et al., 2017; Fleming et al., 2017). Additionally, desomorphine is metabolized by CYP3A4 and UGT, leading to desomorphine glucuronide as the main metabolite (Ventura et al., 2018) while tramadol is metabolized through CYP2B6, CYP2D6, and CYP3A4, with *O*- and *N*-demethylated to five different metabolites. Of these, *O*-desmethyltramadol (desmetramadol) is the most relevant since it has 200 times the μ-affinity of (+)-tramadol. Desmetramadol is metabolized into the active metabolite *N,O*-didesmethyltramadol via CYP3A4 and CYP2B6. The inactive metabolite *N*-desmethyltramadol is metabolized into the active metabolite *N,O*-didesmethyltramadol by CYP2D6 (Gong et al., 2014).

Opioids metabolized through CYP450 or glucuronized can be affected by hepatic disease, and dose reductions may be necessary. Regarding fentanyl, its half-life is not affected significantly in the case of hepatic impairment (Haberer et al., 1982). Nevertheless, dose adjustments are recommended for transdermal fentanyl and, because of its long half-life, its use is not recommended in severe hepatic impairment. The same is applicable to fentanyl pharmaceutical analogs (Food and Drug Administration [FDA], 2018).

Regarding clearance of opioids, while the liver is responsible for the biotransformation of most of them, renal excretion is predominant. Fentanyl excretion in renal impairment is less affected than other opioids such as morphine. With morphine, a significant accumulation of glucuronide metabolites has been observed producing serious adverse effects (Smith, 2009).

## New Synthetic Opioid Drug Interactions

Opioids have a narrow therapeutic index, wide interindividual response variability, and potentially life-threatening toxicity. They can, therefore, lead to clinically relevant DDI.

Their strong potency in comparison to morphine, the prolongation of effects (for some NSO), and their frequent use in combination with other drugs increases the risk of serious drug interactions which can result in toxicity (respiratory depression) and opiate withdrawal symptoms (in the case of previously developed opioid tolerance).

Drug–drug interactions with opioids can be classified in two groups: pharmacodynamic (a) and pharmacokinetic (b) drug interactions.

(a) Pharmacodynamic DDI refer to interactions in which drugs influence each other’s effects directly. In this case, when two drugs are co-administered the concentration-response curve of one or both is altered without a change in the object drug pharmacokinetics (Overholser and Foster, 2011).

Additive interaction means that the effect of the two chemicals is equal to the sum of the effect of the two drugs taken separately, while synergistic interaction means that the effect of two drugs taken together is greater than the sum of their separate effect at the same doses. On the other hand, an antagonistic interaction occurs when the effect of two drugs is actually less than the sum of the effect of the two drugs taken independently.

(b) Pharmacokinetic DDI occur when a drug A (precipitant drug) interferes in the absorption, distribution, metabolism, or excretion of drug B (object drug). Clinically relevant ones mainly affect opioid hepatic metabolism and P-glycoprotein (P-gp). Changes in concentrations due to pharmacokinetic DDI can translate into alterations in opioid effects (increase or reduction of therapeutic or toxic effects).

As most opioids are metabolized by one or more of the CYP450 isozymes (primarily by CYP3A4 and CYP2D6), they can potentially interact with prescription and over-the-counter medication, drugs of abuse, herbal remedies, and dietary supplements that are inducers or inhibitors of them. As an example, CYP3A4 can be induced by garlic and St. John’s Wort while grapefruit inhibits CYP3A4 (Gudin, 2012). In turn, cocaine is a substrate and a weak inhibitor of CYP3A4, and also inhibits CYP2D6, while MDMA is a substrate and an inhibitor of CYP2D6 (O’Mathúna et al., 2008; Lindsey et al., 2012).

When opioids are metabolized active and non-active metabolites appear; the inhibition of this metabolism results in an increase in plasma parent opioid concentration and a reduction of metabolites. The rise in parent drug concentration can lead to an increase of drug therapeutic/toxic effects and intoxication. In the case of inhibiting the metabolism of a pro-drug (only metabolites are active) contrary effects can be observed. Furthermore, if several drugs have the same metabolizing isozyme in common, competitive inhibition can occur among them and result in higher concentrations of one or more. In general, however, if there is no competitive inhibition CYP450 is able to metabolize two substrates of the same isozyme at the same time. Other drugs can inhibit CYP450 isozymes by different mechanisms and even without being substrates. According to the most recent FDA Guidance for Industry of Drug interaction studies, a strong, moderate, and weak inhibitor can increase the area under the curve of concentrations of a sensitive index CYP substrate ≥ 5-fold, ≥2-to < 5-fold, and ≥1.25- to <2-fold, respectively (Food and Drug Administration [FDA], 2017).

Induction of opioid metabolism results in decreased opioid plasma concentrations and may lead to reduced effects (unless the interfered drug is a pro-drug) (Pergolizzi and Raffa, 2017). As previously commented, fentanyl does not have clinically relevant active metabolites, as a result, metabolism inhibition can lead to intoxication and induction to a reduction of effects.

A strong inducer decreases the area under the curve of concentrations of a sensitive index by ≥80%, a moderate one by ≥50% to <80%, and a weak one by ≥20% to <50% (Food and Drug Administration [FDA], 2017). While inhibition can manifest itself immediately, induction requires increased enzyme formation (due to upregulation of enzyme expression) and takes from days to weeks to reach maximum effect and then disappear.

Those opioids metabolized by CYP3A4 have a high risk of DDI (for example, fentanyl), while those metabolized by CYP2D6 have an intermediate risk (for instance, codeine and hydrocodone). CYP2D6 does not respond to induction, consequently, DDI induction with opioids is limited to those metabolized through CYP3A and CYP2B6 (Overholser and Foster, 2011). Clinically relevant interactions are more frequent with strong-moderate inhibitors/inducers than weak ones.

On the other hand, opioids that undergo phase II conjugation, such as morphine, oxymorphone, tapentadol, and hydromorphone, have minimal pharmacokinetic interaction potential. However, interactions can also occur with other drug substrates of enzymes responsible for glucuronidation, such as UGT2B7, or due to polymorphisms in the genes coding those enzymes (Smith, 2009; Gudin, 2012).

In addition, genetic polymorphisms of cytochrome P450 isozymes and genetic variations of the receptors can modulate the effects of drug interactions. As an example, different CYP2D6 metabolizing phenotypes exist: ultrarapid metabolizer, extensive metabolizer, intermediate metabolizer, and poor metabolizer. It has been described that poor metabolizers can have fewer analgesic effects on pro-drugs such as codeine and tramadol which need O-demethylation into morphine, or *O*-desmethyltramadol mediated by CYP2D6, respectively.

As previously commented, pharmacokinetic drug interactions can also occur with drug absorption. P glycoprotein (P-gp) inhibitors (quinidine) can increase concentrations of fentanyl and morphine, while inducers (rifampin) can reduce morphine absorption (Feng et al., 2017).

### Interactions of New Synthetic Opioids With Medication

#### Fentanyl, Morphine, and Pharmaceutical Fentanyl Analogs

Pharmaceutical opioids including morphine, fentanyl, and some fentanyl analogs can interact with a broad spectrum of drugs. **Table 2** depicts DDI described in the product label information of Sublimaze^®^ (fentanyl), Duragesic^®^ (fentanyl), Fentora^®^ (fentanyl), Actiq^®^ (fentanyl), Alfenta^®^ (alfentanil), Rapifen^®^ (alfentanil), Sufenta^®^ (sufentanil), Ultiva^®^ (remifentanil), Avinza^®^ (morphine sulfate), Embeda^®^ (morphine sulfate and naltrexone hydrochloride), and Duramorph^®^ (morphine sulfate) (Food and Drug Administration [FDA], 2018). The table has been completed with information from reference literature on drug interactions (Mozayani and Raymon, 2004; Baxter, 2008; Preston, 2015; Karalliedde et al., 2016).

**Table 2:** Drug–drug interactions with morphine, fentanyl and other pharmaceutical analogs (Mozayani and Raymon, 2004; Baxter, 2008; Preston, 2015; Karalliedde et al., 2016; Food and Drug Administration [FDA], 2018; European Monitoring Centre for Drugs and Drug Addiction [EMCDDA], 2018a).

Opioid	Interacting medication	Type of drug–drug interaction	Result
Alfentanil Fentanyl Morphine Sufentanil	Anticholinergic drugs	PD: Additive or synergistic.	Increase the risk of urinary retention and severe constipation (paralytic ileus).
Fentanyl	Amiodarone	Uncertain.	Profound braycardia, sinus arrest, hypotension.
Fentanyl Morphine	Baclofen	PD: Additive or synergistic.	Enhances analgesic effect, sedation and risk of respiratory depression of opioid
Alfentanil Fentanyl Morphine Remifentanil Sufentanil	Benzodiazepines and other CNS depressants (barbiturates, tranquilizers, anesthetics, antypsychotics, other opioids, alcohol, antihistamines)	PD: Synergistic.	CNS and cardiovascular effects of opioids may be enhanced (hypotension, respiratory depression, profound sedation, coma, death)
Morphine	Cimetidine	PK: Possible decrease in morphine metabolism	Potentiate morphine effects and increase the risk of hypotension, respiratory depression, profound sedation, coma and death.
Alfentanil Fentanyl	Cimetidine	PK: Inhibition of opioid CYP34A mediated metabolism.	Cimetidine reduces opioid clearance, extending the duration of action
Alfentanil Fentanyl Sufentanil	CYP3A4 inhibitors (macrolide antibiotics, azole antifungical agents, HIV- protease inhibitors, grapefruit juice, dialtiazem)	PK: Inhibition of opioid CYP3A4 mediated metabolism.	Increase in plasma concentrations of opioid, increase or prolongation of effects. After stopping inhibitor, concentrations of opioid decrease, resulting in decreased efficacy or withdrawal in patients who have developed physical dependence.
Alfentanil Fentanyl Sufentanil	CYP3A4 inducers (rifampin, carbamazepine, phenytoin)	PK: Induction of opioid CYP3A4 mediated metabolism.	Reduction in plasma concentrations of opioid, decreased efficacy or onset of a withdrawal syndrome. After stopping inducer, concentrations of opioid increase, which could increase or prolong effects or adverse reactions of opioid, and may cause serious respiratory depression.
Fentanyl	Darafenib	PK: Induction of opioid CYP3A4 mediated metabolism.	Possible reduced concentrations of fentanyl.
Alfentanil Fentanyl Morphine Sufentanil	Diuretics	PD: Antagonism.	Reduction of diuretic effects because opioids induce release of antidiuretic hormone.
Morphine	Esmolol	PK: Unknown.	Increase in plasma concentrations of esmolol.
Morphine	Estrogens	PK: Increase metabolism of morphine (glucuronyl transferase).	Effect of morphine may be reduced, requiring higher doses.
Morphine	Ethanol	PK: Inhibition of morphine glucuronidation.	Increase in morphine plasma concentrations, potentially fatal overdose of morphine.
Alfentanil Fentanyl Morphine	Imatinib, nilotinib	PK: CYP3A4 and CYP2D6 inhibition (imatinib). CYP3A4 and P-gp inhibition (nilotinib)	Increase in plasma concentrations of opioid.
Alfentanil Fentanyl Morphine Sufentanil Remifentanil	MAOI (phenelzine, tranylcypromine, linezolid)	PD: Additive or synergistic. PK: MAOI inhibition of opioid metabolism (more common for morphine)	Opioid toxicity (respiratory depression, coma). Potentiation of MAOI effects (hypertension).
Morphine	Metformin	PK: Competition for renal tubular excretion.	Increase in metformin concentrations and risk of lactic acidosis.
Morphine	Metoclopramide	PK: Increase of gastric emptying with metoclopramide.	Increase speed of onset and effect of oral morphine.
Morphine	Mexiletine	PK: Reduction of mexiletine absorption due to a delay in gastric emptying produced by morphine.	Decreased plasma concentrations of mexiletine
Alfentanil Fentanyl Morphine Sufentanil	Muscle relaxants	PD: Additive or synergistic.	Enhancement of muscle blocking action of muscle relaxants, increased degree of respiratory depression.
Fentanyl	Neuroleptics	PD: Additive or synergistic. Unexplained alterations in sympathetic activity following large doses.	Elevated blood pressure.
Alfentanil Fentanyl Sufentanil	Nitrous oxide	PD: Additive or synergistic.	Cardiovascular depression
Alfentanil Fentanyl Morphine Sufentanil Remifentanil	Opioids partial agonists or mixed agonist/antagonists (butorphanol, nalbuphine, pentazocine, buprenorphine)	PD: Antagonism.	Reduction of analgesic effect of full agonists, precipitation of withdrawal.
Opioid agonists	Opioid non-selective antagonists (naloxone, naltrexone, nalmefene)	PD: Antagonism.	Naloxone is indicated for treatment of opioid overdose and naltrexone for opioid use disorders. Antagonists can precipitate opiate withdrawal.
Opioid agonists	Peripherally acting μ-opioid receptor antagonists (methylnaltrexone, naloxegol, alvimopan)	PD: Antagonism	Methylnaltrexone and naloxegol are indicated for reduction of opioid constipation. May produce withdrawal and/or reduction of analgesic effect if blood brain barrier is altered. Alvimopan is indicated to accelerate the time to recovery following partial bowel resection surgery with primary anastomosis.
Morphine	P-glicoprotein inhibitors (quinidine)	PK: Inhibition of P-gp.	Increase the exposure to morphine 2-fold, and can increase risk of hypotension, respiratory depression, profound sedation, coma and death.
Alfentanil Fentanyl Morphine Sufentanil Remifentanil	Serotoninergic drugs (SSRIs, SNRIs (duloxetine), TCAs, MAOI, triptans, 5-HT3 receptor antagonists, mirtazapine, trazodone, tramadol, linezolid, intravenous methylene blue. lythium)	PD: Additive or synergistic.	Serotonin syndrome.
Fentanyl Morphine	TCAs	PK: Inhibition of CYP2D6-fentanyl metabolism. Unknown for morphine.	Increase in opioid plasma concentrations, may increase effects.

*MAOI, monoamine oxidase inhibitors; PK, pharmacokinetic interaction; PD, pharmacodynamic interaction; SNRIs, Serotonin and norepinephrine reuptake inhibitors; SSRIs, selective serotonin reuptake inhibitors; TCAs, tricyclic antidepressants.*

Regarding pharmacodynamic DDI, fentanyl and its pharmaceutical analogs interact with other central nervous system (CNS) sedative drugs such as antihistamines, benzodiazepines, barbiturates, tranquilizers, anesthetics, antipsychotics, and other opioids, increasing their effects.

On the other hand, precipitation of withdrawal can be observed with opioid non-selective antagonists including naloxone, naltrexone, and nalmefene and, in exceptional circumstances, with peripheral μ-opioid antagonists such as methylnaltrexone, naloxegol, and alvimopan (European Medicines Agency [EMA], 2018; Food and Drug Administration [FDA], 2018).

Co-administration of fentanyl with CYP3A4 inducers and inhibitors should be avoided; the same is applicable for pharmaceutical fentanyl analogs. Morphine, in turn, is mainly metabolized through UGT2B7 and minimal pharmacokinetic changes have been described with UGT inhibitors (Overholser and Foster, 2011).

Opioid-induced constipation is predominantly mediated by gastrointestinal μ-opioid receptors. Selective blockade of these peripheral receptors might relieve constipation without compromising the centrally mediated effects of opioid analgesia or precipitating withdrawal. Two of these drugs are methylnaltrexone (Relistor^®^) and naloxegol (Movertig^®^), approved by the EMA (European Medicines Agency [EMA], 2018). Patients with blood-brain barrier alteration may have opioid withdrawal symptoms or experience reduction in the analgesic effects of opioids when using these peripheral antagonists. Alvimopan (Entereg^®^) is another peripheral antagonist approved by the FDA, indicated in this case for treatment of post-operative ileus induced by opioid agonists (Food and Drug Administration [FDA], 2018).

In abuse-deterrent oral preparations, a mixture of an agonist (oxycodone, buprenorphine) and an antagonist (naloxone, naltrexone) is employed in order to reduce the abuse of these combinations by intravenous route. An antagonist is added to be released upon manipulation and interfere with, reduce, or defeat the euphoria associated with abuse. For instance, naloxone is poorly absorbed when taken orally or sublingually and it is added to decrease the risk that the medication will be misused through injection (Papaseit et al., 2013; Argoff et al., 2014; Lee et al., 2017).

#### Non-pharmaceutical Fentanyl Analogs and Other NSO

Taking into account the DDI described for fentanyl and its pharmaceutical analogs, some interactions with other NPS opioids can be anticipated.

Theoretically, pharmacodynamic interactions can be observed with non-medical NSO and benzodiazepines and other CNS depressants (barbiturates, opioids, tranquilizers, anesthetics, antipsychotics, and antihistamines) due to their synergistic effects on CNS depression. Antagonistic effects can appear with NPS opioid-full agonists and other opioid-mixed agonists or mixed agonists/antagonists, and also with full antagonists, for instance, naloxone, naltrexone, and nalmefene.

Insufficient information is available about the metabolism of these substances, as a result, the pharmacokinetic interactions are more difficult to predict. In general, precaution is mandatory when NSO are administered with CYP3A4/CYP2D6 inhibitors and CYP3A4 inducers.

### Interactions of New Synthetic Opioids With Drugs of Abuse

Few experimental human data are available regarding interactions of NPS opioids with other drugs of abuse.

In general, the association of other opioids with heroin and alcohol increases CNS depression which can lead to serious side effects including respiratory distress, coma, and even death (Food and Drug Administration [FDA], 2018).

Frequently opiate abuse includes combinations with stimulants (“speedball” or “bombeta”). The most popular mixture among drug injectors is heroin with cocaine or amphetamines in the same syringe. A new trend is fentanyl-laced cocaine (adding fentanyl to cocaine) for the purpose of speedballing, to combine the rush of the stimulant (cocaine) with a drug that depresses the CNS (fentanyl) thus helping to ease the after effects. Furthermore it should be noticed that most users are inadvertently exposed to fentanyl or other NSO when cocaine or other drugs of abuse are laced with them. In this cases, a drug interaction can also occur and the user can have unexpected adverse effects.

Amphetamines when combined with opiates enhance the sense of euphoria (Atkinson and Fudin, 2014). Dexamphetamine and methylphenidate increase the analgesic effects of morphine and other opioids and reduce their sedative and respiratory depressant effects (Mozayani and Raymon, 2004; Baxter, 2008).

Additionally, amphetamine-like drugs with certain opioids may increase the risk of serotonin syndrome through effects on the serotonin transporter or serotonin receptors. Among reported cases, tramadol and fentanyl are the most frequently involved, while morphine is merely anecdotal and there are no cases with heroin (Rickli et al., 2018).

#### Heroin, Fentanyl, and Morphine

Heroin can interact with other drugs of abuse such as alcohol and psychostimulants. The combination of heroin and alcohol produce a sensation of greater drug pleasure and stronger “high” than the two drugs on their own. Furthermore, the inhibition of heroin metabolism by high doses of ethanol is suggested due to an increase in free morphine in the blood/total morphine in blood ratio (Polettini et al., 1999). In fact, ethanol inhibits two steps of the heroin metabolism, the hydrolysis of 6MAM to morphine, and the glucuronidation of morphine to morphine-3-glucuronide and morphine-6-glucuronide (Thaulow et al., 2014).

Heroin enhances cocaine-rewarding effects (Hemby et al., 1999) and reduces cocaine- induced anxiety or agitation while cocaine tempers opiate-induced sedation. In this case, a pharmacodynamic interaction seems to prevail as no changes in the ratio morphine in blood/total morphine in blood have been found (Polettini et al., 2005).

The combination of heroin and methamphetamine has been reported to produce a significant “high.” Enhanced rewarding effects and higher stimulation of behavior than each drug on its own have been reported in animal models (Ranaldi and Wise, 2000; Trujillo et al., 2011). D-amphetamine increases the effects of heroin. In fact, opiate abusers employ amphetamines to increase the effects of poor quality heroin (Mozayani and Raymon, 2004).

Serious interactions can also occur between fentanyl combined with cocaine, heroin, and alcohol. At a pharmacodynamic level, fentanyl and alcohol exert a synergic depressive action on the cardio-circulatory system while cocaine does the opposite, exciting it, constricting the arteries, and inducing hyperkinetic cardiac arrhythmia (Ferrara et al., 1994). Furthermore, the use of alcohol with fentanyl may increase nervous system side effects such as drowsiness, dizziness, lightheadedness, difficulty concentrating, and impairment in thinking and judgment. In severe cases, low blood pressure, respiratory distress, fainting, coma, or even death may occur (Food and Drug Administration [FDA], 2018).

A recent study conducted in rats demonstrates that the effects of heroin plus fentanyl on brain oxygenation and temperature are enhanced when compared to the effects of either drug alone, providing evidence for synergism (Solis et al., 2018).

No human experimental studies have been identified studying the interaction of fentanyl and psychostimulants (cocaine, methamphetamine, and amphetamine).

Regarding morphine, it administration with cocaine in healthy volunteers increased cardiovascular and subjective effects, but less than predicted, and no changes were found in blood concentrations (Foltin and Fischman, 1992). In patients with postoperative and chronic malignant pain, the administration of cocaine with morphine did not increase analgesic response. Interaction effects were observed in terms of positive changes (cheerful, friendly) in postoperative patients, but negative ones (sad, serious) in patients with chronic pain (Kaiko et al., 1987).

Cannabis enhanced the effects of morphine in chronic pain (Lynch and Clark, 2003), smokers (nicotine), however, may require more opioid (morphine, fentanyl) analgesics for postoperative pain than non-smokers. It has been suggested that smoking may have an effect on pain perception and/or opioid response (Baxter, 2008).

On the other hand, administration of morphine with amphetamine to healthy volunteers showed an increase in opiate symptoms, liking and euphoria scale scores which were greater than the effects of either drug alone. Physiological effects were mutually antagonistic in pupillary effects, respiratory rate, temperature and pulse rate, though morphine had little or no effect on the amphetamine-induced blood pressure increases. The combination has a greater potential to be abused because of the additive euphoria and a lessening of side effects (Jasinski and Preston, 1986).

The use of two or more opioid μ-agonists can increase pharmacological and toxic effects whilst a μ-agonist with a μ-antagonist can reduce them. In fact, naloxone is the treatment of choice to address acute opioid toxicity. The simultaneous administration of a μ-agonist (such as fentanyl, morphine, or heroin) and a partial agonist (buprenorphine) or an antagonist can precipitate a withdrawal in addicted subjects. This can be observed with non-selective antagonists including naloxone, naltrexone, and nalmefene and, in exceptional circumstances, with peripheral mu-opioid antagonists such as methylnaltrexone, naloxegol, and alvimopan (European Medicines Agency [EMA], 2018; Food and Drug Administration [FDA], 2018).

#### Pharmaceutical, Non-pharmaceutical Fentanyl Analogs, and Other NSO

Interactions between NPS opioids, other than fentanyl, and drugs of abuse can be extrapolated from those described for morphine, fentanyl, and heroin. Therefore, serious pharmacodynamic interactions can be anticipated between NSO mixed with heroin, alcohol, cocaine and other psychostimulants, and also with opioid antagonists.

Additionally, potential pharmacokinetic interactions can occur, with unknown clinical relevance, between cocaine, methamphetamine or MDMA, and NSOs using CYP2D6 as the main metabolic route. As previously mentioned, cocaine is a strong inhibitor of CYP2D6 (Lindsey et al., 2012) while MDMA acts as a high affinity substrate and a potent mechanism-based inhibitor of CYP2D6 (O’Mathúna et al., 2008). In turn, methamphetamine *N*-demethylation leading to amphetamine, and aromatic hydroxylation producing 4-hydroxymethamphetamine, are partially regulated by CYP2D6 (de la Torre et al., 2012).

## Clinical Assessment and Treatment

General recommendations to address the current opioid crisis are based on three main measures. The first is to reduce the supply of both licit and illicit opioids, the second to increase accessibility to evidence-based treatment of opioid use disorders (decrease the demand), and the last to provide overdose reversal medication (Schnoll, 2018).

Focusing on the misuse of non-prescribed opioids, over 20% of patients on chronic opioid therapy have a opioid use disorder (Boscarino et al., 2015). To reduce the risks associated with long-term opioid therapy, including opioid use disorder and overdose, several recommendations are available. Centers for Disease Control and Prevention guidelines for prescribing opioids for chronic pain advise clinicians to continue therapy only if there is a clinically meaningful improvement in pain and function that outweighs risks (Dowell et al., 2016).

There are some factors that increase the risk of developing addiction to analgesic opioids. They are related to individual aspects and the pharmacological characteristics of opioids. Among the individual factors are the following: (a) genetics: some variants in opioid receptor genes OPRM1, OPRK1, and OPRD1 and preproenkephalin that modulate the perception of pain, can increase the risk of further development of addiction (Khokhar et al., 2009); (b) a history of depression, anxiety (panic, social phobia, agoraphobia, posttraumatic stress disorder) and other substance use disorders (alcohol, benzodiazepines, cannabis, cocaine); (c) sociodemographics: women are at greater risk in relation to emotional and affective problems (e.g., depression), while men are at greater risk in relation to problematic and/or illegal behavior; also lower age is linked to more risk; (d) perception of pain: patients with more subjective pain, multiple complaints of pain, and greater limitations related to pain, have more risk. Finally, as previously described, among the pharmacological characteristics of opioids their fast absorption and/or short half-life multiply the risk of misuse. Although none of these factors alone increases the risk of aberrant behavior in a particular individual, when occurring simultaneously they can play a major role.

It is crucial to employ immediate-release opioids when commencing treatment instead of extended-release/long-acting opioids, because in the last formulations there are higher amounts of opioids. There is, however, contradictory information comparing short-acting and long-acting opioids due to different dosages administered (Chou et al., 2015). The lowest effective dosage should be prescribed. For chronic pain clinicians should evaluate benefits and harm within 1–4 weeks of initiation or dose escalation of treatment, and at least every 3 months after that. When opioids are used for acute pain, again the lowest effective dose of immediate-release opioids should be used, for 3 days or less in most occasions (Dowell et al., 2016). Other recommendations when prescribing opioids for pain treatment in patients with a history of substance use disorders, are to prescribe tamper-deterrent formulations (e.g., crush-resistant tablets) or combinations of ingredients (combination of agonist/antagonist), the use of a sequestered aversive agent, a pro-drug, and a novel delivery system (Papaseit et al., 2013; Argoff et al., 2014; Lee et al., 2017). There are contradictory results regarding the type of opioid prescribed, as some reviews recommend prescribing weaker ones, such as tramadol (Chou et al., 2015), other authors, however, found a higher risk of abuse with this type of medication compared to strong opioids (Higgins et al., 2018). Several tools are available that can help identify patients at risk. They include the following: the Opioid Misuse Measure (Melzer et al., 2011), the Opioid Misuse Measure (COMM) (Butler et al., 2010), the Opioid Risk Tool (Webster and Webster, 2005), the Screener and Opioid Assessment for Patients with Pain (Butler et al., 2008), and the Brief Risk Interview (Wu et al., 2006).

Furthermore, there are monitoring strategies to assess problematic use (misuse, abuse, addiction) in such patients including urine drug testing, medication counts, prescription drug monitoring programs, and blood level monitoring although their effectiveness has not yet been well-established (Voon et al., 2017).

### Treatment of Overdoses: Naloxone

Regarding opioid overdose, clinical presentation is recognized by the classic opioid triad typically characterized by pinpoint pupils, unconsciousness, and respiratory depression which can lead to brain damage or even death. Currently, naloxone, a short-acting, broad opioid receptor antagonist, is the only useful pharmacological treatment when administered shortly after overdose (Boyer, 2012). In low doses naloxone can reverse opioid side effects without significantly reversing analgesia. At high doses, however, naloxone can block opioid analgesia causing precipitated opioid withdrawal (Levine et al., 1979).

Naloxone is available as a solution for intravenous, intramuscular, subcutaneous, and orotracheal injection, and as a spray for nasal administration (Narcan^®^ Nasal Spray and Nyxoid^®^) as an alternative to intramuscular or subcutaneous auto-injection (Evzio^®^) (Food and Drug Administration [FDA], 2018; European Medicines Agency [EMA], 2018). The manufacturers recommended an initial dose of 2–4 mg intranasally or 0.4–2 mg intramuscularly/subcutaneously to be repeated after 2–3 min as needed. There is, however, no consensus or recommendation on which of the doses should be selected in a given case of opioid overdose. Because the half-life and the duration of naloxone effects after administration are short there is a risk of recurrence of respiratory depression or inadequate response following reversal with naloxone when treating the effects of long-acting, high-dose or potent NSOs (Rzasa Lynn and Galinkin, 2018). Naloxone should be administrated at a dose of 0.04 mg intravenously with upward titration to 10 mg for subjects with opioid-induced respiratory compromise (Hoffman et al., 2015). There is little evidence that naloxone doses required to treat fentanyl, its analogs and other NSO overdoses might be higher. In fact, empirical data from previous fentanyl epidemics in the US show that doses of naloxone up to 12 mg were required to rescue subjects intoxicated with fentanyl (Schumann et al., 2008).

American Heart Association (2015) incorporated new concepts regarding opioid overdose management, education, and naloxone training and distribution. It included empiric administration of intramuscular/intranasal naloxone to all unresponsive subjects of possible opioid-associated life-threatening emergencies as an adjunct to standard first aid and non-healthcare provider basic life support protocols. For patients with known or suspected opioid overdose presenting a definite pulse but no normal breathing or only gasping (i.e., a respiratory arrest), intramuscular or intranasal naloxone empiric administration by appropriately trained rescuers is also reasonable. Responders should not delay access to more advanced medical services while awaiting the patient’s response to naloxone or other interventions (American Heart Association, 2015).

Additionally, nalmefene, an opioid antagonist analog of naltrexone now discontinued, was also approved through injection (Revex^®^) by the FDA for the management of known or suspected opioid overdose (Food and Drug Administration [FDA], 2018). At this moment it is approved orally by the EMA (Selincro^®^) to reduce alcohol consumption in alcohol dependence (European Medicines Agency [EMA], 2018).

Other possible drugs or strategies under research to treat respiratory depression are 5-hydroxytryptamine type 1A agonists, ampakines, and phrenic-nerve-stimulation devices. In addition, technology to detect overdose and autoinjected naloxone is being studied (Volkow and Collins, 2017).

### New Synthetic Opioid Use Disorders and Treatment

At present, effective pharmacological treatment for opioid use disorders to reduce cravings and withdrawal symptoms include methadone (agonist) and buprenorphine (partial agonist), both of which are listed by the World Health Organization (WHO) as essential medicines, and naltrexone (an opioid antagonist) that blocks the effects of opioids (Volkow, 2018; Volkow et al., 2018). In addition, methadone and buprenorphine have been shown to increase adherence to antiretroviral therapy in HIV-infected drug users and treatment retention of opioid-dependent pregnant women (Bisaga et al., 2018). They are available as extended-release, transdermal, and long-lasting formulations (e.g., extended-release naltrexone, buprenorphine implant, long-acting injectable naltrexone).

Regarding psychosocial interventions, there is a lack of evidence on the efficacy of concurrent psychotherapy (mindfulness and cognitive behavioral treatments) in the management of chronic pain and opioid misuse (Eilender et al., 2016).

To combat the opioid crisis, besides optimizing the treatment of overdoses and continuing advances in the field of abuse deterrent formulations (10 approved by the FDA: 3 oxycodone, 1 oxycodone + naloxone, 1 oxycodone + naltrexone, 2 morphine, 1 morphine + naltrexone, and 2 hydrocodone products) the development of new pharmacological medication and strategies to manage opioid use disorders is crucial (Becker and Fiellin, 2017).

Current research is focused on brain-stimulation technologies, vaccines, and monoclonal antibodies, together with other drugs such as lorcaserin (5-HT2c antagonist) that have been shown to reduce opioid seeking in rodents, and lofexidine (α2A-adrenergic receptor agonist) to control withdrawal symptoms (Volkow and Collins, 2017).

## Conclusion

New synthetic opioids are frequently used with other substances such as illegal drugs and medication. Their combination can lead to clinically relevant interactions and serious intoxications. Pharmaceutical fentanyl analog interactions with CYP3A4 inducers and inhibitors have been described. Pharmacokinetic interactions with non-medical fentanyl analogs and new compounds are, however, difficult to predict because there are limited data regarding their pharmacokinetics and metabolism. On the other hand, pharmacodynamic interactions between NSO and other drugs acting on the CNS can be anticipated. Effects are expected to be similar to those reported for heroin, morphine, and fentanyl, as they also act mainly as μ-opioid receptor agonists. In the case of opioid overdose due to a DDI, naloxone can be used as an antidote, taking into account the fact that the required doses might be higher than for traditional opioids.

## Author Contributions

CP-M and EP wrote the manuscript that was reviewed by FF, AF, MT, and MF.

## Conflict of Interest Statement

The authors declare that the research was conducted in the absence of any commercial or financial relationships that could be construed as a potential conflict of interest.
